# Multiple Regions Drive Hepatitis Delta Virus Proliferation and Are Therapeutic Targets

**DOI:** 10.3389/fmicb.2022.838382

**Published:** 2022-04-06

**Authors:** Jun Zi, Xiuzhu Gao, Juan Du, Hongqin Xu, Junqi Niu, Xiumei Chi

**Affiliations:** ^1^Gene Therapy Laboratory, Center for Pathogen Biology and Infectious Diseases, First Hospital of Jilin University, Changchun, China; ^2^Department of Hepatology, Center for Pathogen Biology and Infectious Diseases, First Hospital of Jilin University, Changchun, China; ^3^Institute of Virology and AIDS Research, First Hospital of Jilin University, Changchun, China

**Keywords:** hepatitis delta virus, single-stranded RNA, antigen, viral structures, virus life cycle, therapy

## Abstract

Hepatitis Delta Virus (HDV) is the smallest mammalian single-stranded RNA virus. It requires host cells and hepatitis B virus (HBV) to complete its unique life cycle. The present review summarizes the specific regions on hepatitis D antigen (HDAg) and hepatitis B surface antigen (HBsAg) that drive HDV to utilize host cell machinery system to produce three types of RNA and two forms of HDAg, and hijack HBsAg for its secretion and *de novo* entry. Previously, interferon-α was the only recommended therapy for HDV infection. In recent years, some new therapies targeting these regions, such as Bulevirtide, Lonafarnib, Nucleic acid polymers have appeared, with better curative effects and fewer adverse reactions.

## Introduction

Hepatitis delta virus is a defective virus that requires HBV to proliferate in human hepatocytes. A recent meta-analysis estimated that 292.0 (251.5–341.1) million individuals were HBsAg positive, corresponding to a global prevalence of 3.9% (3.4–4.6) (Polaris Observatory Collaborators, 2018); another meta-analysis showed that 0.16% (0.11–0.25) of global population, totaling 12.0 (8.7–18.7) million people, were estimated to be anti-HDV positive (Stockdale et al., 2020). HDV prevalence varies according to geography (Table 1).

**TABLE 1:** Estimates of HDV prevalence in the general population (95% CI) [Source: Stockdale et al. (2020)].

	AFR	AMR	EMR	EUR	SEAR	WPR	Global
Anti-HDV prevalence,%	0.36	0.04	0.12	0.05	0.06	0.25	0.16
HDV RNA prevalence,%	0.15	0.03	0.06	0.03	0.03	0.19	0.09

*AFR, African Region; AMR, Region of the Americas; EMR, Eastern Mediterranean Region; EUR, European Region; SEAR, South-East Asian Region; WPR, Western Pacific Region.*

Hepatitis delta virus infection occurs *via* two ways: coinfection and super-infection. HBV and HDV simultaneously co-infect healthy people, causing extensive hepatic necrosis and severe fulminant hepatitis with a high fatality rate. Most adults can eliminate both viruses and recover from coinfection, and < 5% of cases develop chronic hepatitis D (CHD) (Botelho-Souza et al., 2017). Compared with HBV mono-infection and two viruses’ coinfection, chronic hepatitis B patients with HDV super-infection face an accelerated disease progression to cirrhosis and an increased risk of developing hepatocellular carcinoma. Usually, such patients cannot clear HDV, and 90% of cases develop CHD (Fattovich et al., 2000).

Hepatitis B virus vaccine protects healthy individuals from HBV and HDV infections. However, there is currently no HDV vaccine available protecting chronic hepatitis B patients from HDV infection. Before the European Medicines Agency approved the conditional marketing authorization of Bulevirtide in July 2020, interferon (IFN)-α was the only therapy for CHD recommended by professional and societal guidelines. However, IFN-α can lead to common and sometimes severe adverse reactions, with a high recurrence rate (Abbas et al., 2011).

We summarize the specific regions on HDAg and HBsAg that drive HDV proliferation, and the curative effect of some therapies targeting these regions. We further list some mechanisms underlying the HDV life cycle that are yet ill-understood, and some regions that are potential therapeutic targets. Investigating HDV proliferation mechanisms and the functions of specific regions on HDAg and HBsAg will help us to develop new treatment strategies associated with better therapeutic effects, fewer adverse reactions, and lower recurrence rates.

## Hepatitis Delta Virus Structure

Hepatitis delta virus is 35–37 nm in diameter and composed of HDV ribonucleoprotein (RNP) complex and HBsAg-containing envelope (Bonino et al., 1981). HDV RNP complex consists of HDV genome RNA (gRNA) and HDAg (Ryu et al., 1993).

### Hepatitis Delta Virus Genome RNA

**FIGURE 1:**
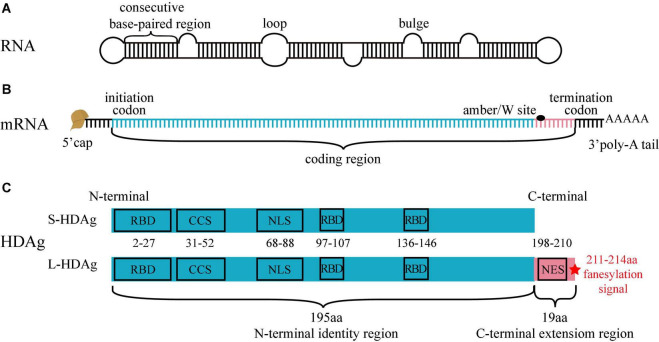
Structure of HDV RNA, mRNA and HDAg. **(A)** HDV RNA is a circular single-stranded RNA, 74% ribonucleotides form consecutive base-paired regions, interspersed by small loops or bulges formed by the unpaired ribonucleotides. All ribonucleotides fold into a quasi-double-stranded, unbranched rod-like structure. **(B)** There are a 5′ cap, a 3′ poly-A tail, and a coding region in HDV mRNA. The ribonucleotides from initiation codon to amber/W site (blue segment) encodes S-HDAg and L-HDAg N-terminal identity region, and the ribonucleotides from amber/W site to termination codon (pink segment) encodes L-HDAg C-terminal extension region. **(C)** S-HDAg and L-HDAg are characterized by three RNA-binding domains (RBD), a coiled-coil sequence (CCS), a nuclear localization signal (NLS) in the N-terminal identity region (blue box), and a nuclear export signal (NES), a farnesylation signal (red star) in the C-terminal extension region (pink box).

There are currently eight HDV genotypes (HDV-1–8), the ribonucleotide number of HDV gRNA is between 1,672 and 1,697 (genotype-dependent) (Wang et al., 2021). HDV gRNA is a circular single-stranded RNA of negative polarity, and about 74% of ribonucleotides form dozens of consecutive base-paired regions (9–15 bp), interspersed with small internal loops or bulges formed by the unpaired ribonucleotides. All ribonucleotides fold into a quasi-double-stranded (ds), unbranched, rod-like structure (Casey, 2012; Lempp et al., 2016; Hsu et al., 2019; Figure 1A).

Genomic strand ribozyme and antigenome strand ribozyme are the only viral enzymes possessed by HDV, display related sequences and are similar in the predicted secondary structure, and both are crucial for HDV RNA replication (Riccitelli and Lupták, 2013). HDV gRNA only encodes two proteins in the same open reading frame (Xia et al., 1990).

### Hepatitis D Antigen

Two forms of HDAg that are translated from HDV mRNA have been identified, including small-HDAg [S-HDAg with 195 amino acids (aa), except HDV-3, -4, and -6 with 194 aa] and large-HDAg (L-HDAg with 214 aa, except HDV-4, -6 with 213 aa). HDV mRNA structure is similar to that of the host mRNA, containing a 5′ cap, a 3′ poly-A tail, and a coding region (Taylor, 2015). A variable amber/W site on the coding region regulates the production of HDAg. The ribonucleotides from initiation codon to amber/W site encode S-HDAg and L-HDAg N-terminal identity region (195 or 194 aa), and the ribonucleotides from amber/W site to termination codon encode L-HDAg C-terminal extension region (19 or 20 aa). Genotypes of HDV except HDV-3, 4, and 6 are described in the following text (Lempp et al., 2016; Figure 1B).

The three critical regions on the S-HDAg and L-HDAg N-terminal identity region (1–195 aa) are as follows: (i) RNA-binding domain (RBD, 2–27 aa, 97–107 aa, and 136–146 aa), binding to HDV RNA to form HDV RNP (Lee et al., 1993; Poisson et al., 1993); (ii) Coiled-coil sequence (CCS, 31–52 aa), mediating the polymerization of HDAg (Xia and Lai, 1992); (iii) Nuclear localization signal (NLS, 68–88 aa), driving the translocation of HDAg from cytoplasm to nucleus (Xia et al., 1992). The two critical regions on the L-HDAg C-terminal extension region (196–214 aa) are as follows: (i) Nuclear export signal (NES, 198–210 aa), driving the translocation of HDV RNP from nucleus to cytoplasm (Lee et al., 2001); (ii) Farnesylation signal (211–214 aa), located at the last four amino acids of the N-terminal and covalently linked to farnesyl lipid group, which inhibits HDV RNA amplification and promotes progeny viruses assembly (Glenn and White, 1991; Hwang and Lai, 1993; Figure 1C).

### Hepatitis Delta Virus Ribonucleoprotein Complex

**FIGURE 2:**
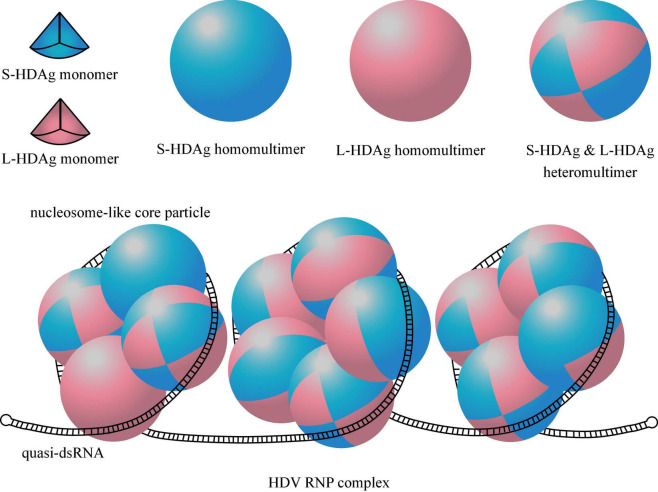
Structure of HDAg multimers and HDV ribonucleoprotein (RNP) complex. One eighth of the sphere represents a HDAg monomer, the blue one represents S-HDAg, the pink one represents L-HDAg. A HDAg octamer containing 8 S-HDAg or 8 L-HDAg is called homomultimer, and is called heteromultimer when it contains a mix of S-HDAg and L-HDAg. Quasi-double-stranded RNA (quasi-dsRNA) wrap around four or five HDAg octamers as a nucleosome-like core particle, and the whole HDV RNP contain 2–6 such core particles.

Hepatitis delta virus RNP complex is 18.7 ± 2.5 nm in diameter and composed of HDV gRNA and 70–200 HDAg monomers (Ryu et al., 1993; Gudima et al., 2002). Before binding to RNA, HDAg monomers are subjected to antiparallel coiled-coil mediated dimerization *via* CCS (Zuccola et al., 1998). These HDAg dimers continue to polymerize into HDAg octamers that exist as either S-HDAg/L-HDAg homomultimers or S-HDAg and L-HDAg heteromultimers (Xia and Lai, 1992; Figure 2). The number of S-HDAg and L-HDAg monomers is equivalent in circulating HDV (Bergmann and Gerin, 1986).

The loops and bulges contribute to the flexibility of the quasi-dsRNA to wrap around HDAg multimers *via* arginine-rich, positively charged RBD for optimal compression. Although the exact assembly structure of dsRNA and HDAg is not yet clear, it is speculated that quasi-dsRNA wraps around four or five HDAg octamers to form a nucleosome-like core particle (Abeywickrama-Samarakoon et al., 2020). The presence of about 70–200 HDAg monomers per HDV implies 2–6 such nucleosome-like core particles per HDV RNP (Griffin et al., 2014; Figure 2).

### Hepatitis B Surface Antigen Containing Envelope

**FIGURE 3:**
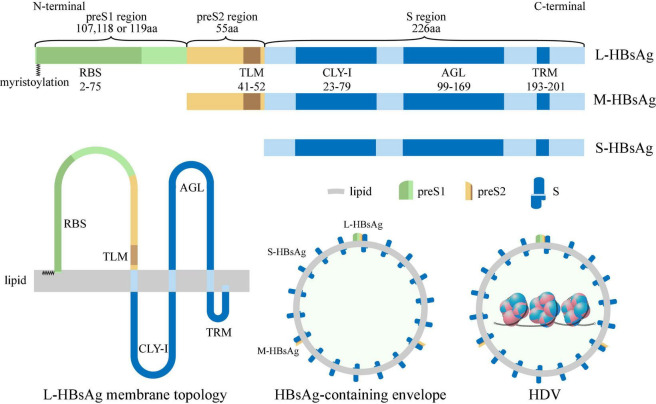
Structure of HBsAg, HBsAg-containing envelope and HDV. S-HBsAg contains S region (blue box), M-HBsAg contains preS2 (yellow box) and S region, and L-HBsAg contains preS1 (green box), preS2 and S region, and the N-terminal of L-HBsAg is myristoylated (zigzag line). Receptor-binding site (RBS) located at preS1 region; Translocation motif (TLM) located at preS2 region; Cytosolic loop I (CYL-I), antigenic loop (AGL), and try-rich motif (TRM) located at S region. The HBsAg-containing envelope of HDV is a host-derived outer surface lipid coat containing three forms of HBV surface antigens.

The HBsAg-containing envelope is a host-derived outer surface lipid coat containing three forms of HBsAgs (Steiner et al., 1974), including small-HBsAg (S-HBsAg, 226 aa), middle-HBsAg (M-HBsAg, 281 aa), and large-HBsAg (L-HBsAg, 388–400 aa, genotype-dependent). All these antigens are encoded by a single open reading frame divided by three in frame standing initiation codons, resulting in preS1 (107, 118, or 119 aa, genotype-dependent), preS2 (55 aa), and S (226 aa) translation regions respectively. S-HBsAg contains the S region, M-HBsAg contains the preS2 and S region, and L-HBsAg contains the preS1, preS2, and S region (Jiang and Hildt, 2020). In the N-terminal of L-HBsAg, a myristoyl group linked to Gly-2 (Bruss et al., 1996; Figure 3). Two promoter sequences regulate the production of HBsAg. The upstream preS1 promoter gives rise to 2.4 kb transcripts that are translated mainly into the L-HBsAg, while the downstream preS2/S promoter promotes the transcription of 2.1 kb mRNAs that are translated into the M-HBsAg and S-HBsAg (Siddiqui et al., 1986). The preS1 promoter is negatively regulated by preS2/S promoter, M-HBsAg and S-HBsAg are produced in excess relative to the L-HBsAg (Bulla and Siddiqui, 1989). The HBsAg-containing envelope of HDV comprises of approximately 95% S-HBsAg, 5% M-HBsAg, and 1% L-HBsAg (Bonino et al., 1986).

Also, several critical regions have been identified in HBsAg (Yang et al., 2021), including (i) receptor-binding site (RBS, 2–75 aa of the myristoylated preS1 region), binding to specific receptors during *de novo* entry (Le Duff et al., 2009); (ii) translocation motif (TLM, 41–52 aa of the preS2 region), driving translocation of HDV RNP across the HBsAg-containing envelope into the cytoplasm during *de novo* entry (Stoeckl et al., 2006); (iii) cytosolic loop I (CYL-I, 23–79 aa of the S region), which is crucial for envelopment (Löffler-Mary et al., 2000); (iv) antigenic loop (AGL, 99–169 aa of the S region), determining the antigenicity and attaching to the cell membrane during *de novo* entry (Sureau and Salisse, 2013); (v) Try-rich motif (TRM, 193–201 aa of the S region), binding to HDV RNP during HDV assembly (Komla-Soukha and Sureau, 2006; Figure 3).

## Hepatitis Delta Virus *de novo* Entry

The *de novo* entry of HDV relies on the HBsAg-containing envelope, and the mechanism underlying *de novo* entry is also similar to that in HBV infection. HDV primarily targets human hepatocytes, and no evidence has shown that HDV naturally infects other cells (Negro et al., 1989). HDV can be experimentally delivered into, and spontaneously replicate in a wide range of non-hepatic cells, such as mouse brain, kidney, testis, skeletal muscle cells (Polo et al., 1995), and Hela cells (Greco-Stewart et al., 2009). This proves that the hepatotropism of HDV is the tropism of *de novo* entry.

The *de novo* entry of HDV is mainly divided into three steps, including attachment, recognition, and entry into cells, and these processes rely on S region, preS1 region, preS2 region of HBsAg, respectively. L-HBsAg is necessary for *de novo* entry as it provides the only source of preS1 region for recognition.

### Attachment

At the beginning of the *de novo* entry, the AGL on the HBsAg S region, which is exposed on the surface of the HBsAg-containing envelope, attaches to the heparan sulfate proteoglycans (HSPG), which is expressed on the outer face of the hepatocyte membrane (Sureau and Salisse, 2013). One particular HSPG, glypican-5, is an entry factor, but it is not necessary for *de novo* entry, as its abrogation is insufficient to completely prevent infection (Verrier et al., 2016).

### Recognition

Attaching to low-affinity HSPG is important but not sufficient for HDV to enter into hepatocytes, as it should be further recognized by high-affinity specific receptor [human sodium taurocholate coreceptor peptide (hNTCP)] (Yan et al., 2012). Through interacting with E-cadherin, hNTCP is distributed on the basolateral membrane of differentiated hepatocytes and responsible for bile acid uptake (Hu et al., 2020). The bile acid-binding region of hNTCP interacts with the RBS on the myristoylated N-terminal of L-HBsAg, allowing HDV to enter into hepatocytes (Meier et al., 2013). Unlike dispensable glypican-5, hNTCP is essential for the *de novo* entry. Knockdown of hNTCP in HepaRG cells *via* small hairpin RNA prevents them from HDV infection (Ni et al., 2014). Primary hepatocytes of mouse, rat, dog, pig, rhesus, and cynomolgus macaque are resistant to HDV infection, but after being transduced with adeno-associated viral vectors encoding hNTCP, these cells are susceptible to HDV infection (Lempp et al., 2017).

### Entry Into Cells

The specific entry mechanism is not yet clear. However, considering that HDV has the same envelope as HBV, it might follow an entry process that is identical or very similar to that used by HBV. After binding with L-HBsAg, hNTCP is markedly oligomerized (Fukano et al., 2018). Then, triggered by epidermal growth factor receptor (Iwamoto et al., 2019), the cell membrane fuses and internalizes virus-hNTCP complex *via* clathrin-mediated endocytosis pathway (Herrscher et al., 2020). Epidermal growth factor receptor is also internalized in the virus-carried vesicle, triggering the relocation of the vesicle to the Rab5-containing early endosome and then to the Rab7-containing late endosome (Macovei et al., 2013; Iwamoto et al., 2020). Endosomal proteases expose TLM on HBsAg preS2, and the unmasking TLM drives translocation of HBV core particles or HDV RNP across the endosomal membrane into the cytoplasm (Stoeckl et al., 2006), but deleting TLM does not interfere with the infection (Blanchet and Sureau, 2007; Gudima et al., 2007).

After being released into the cytoplasm, HBV core particles seem to be delivered to the nucleus *via* microtubule-mediated transportation process (Rabe et al., 2006). HDV RNP is also transported to the nucleus, a process driven by NLS on HDAg (Tavanez et al., 2002). Supposedly, entry of a single RNP into the nucleus is sufficient for HDV infection (Macnaughton and Lai, 2006).

## Hepatitis Delta Virus RNA Amplification

Hepatitis delta virus RNA is amplified in the nucleus and the amplification does not require the presence of HBV (Giersch et al., 2019). HDV does not express its RNA-dependent RNA polymerase or use HBV polymerase, rather uses the host’s DNA-dependent RNA polymerase for RNA amplification. Accumulating evidences have indicated that RNA polymerase I (Pol-I) and RNA polymerase II (Pol-II) are associated with the extremities of both polarities of HDV RNA and involved in amplification. Although RNA polymerase III (Pol-III) is also associated with HDV RNA and seems to be involved in amplification, its exact function is not yet clear (Greco-Stewart et al., 2009).

Hepatitis delta virus RNA amplification involves three types of HDV RNA, including genomic RNA (gRNA, ∼1,700 nt), antigenomic RNA (agRNA, ∼1,700 nt), and mRNA (∼800 nt). The copy number of these RNA is approximately 300,000, 60,000, and 600/cell, respectively (Taylor, 2015). Moreover, gRNA and agRNA bind with HDAg to form gRNP or agRNP, respectively. The three steps of RNA amplification are as follows: (i) In the nucleoplasm, Pol-II uses gRNP as a template to transcribe mRNA; (ii) In the nucleolus, Pol-I uses gRNP as a template to replicate agRNA; (iii) In the nucleoplasm, Pol-II uses agRNP as a template to replicate gRNA (Macnaughton and Lai, 2006; Abeywickrama-Samarakoon et al., 2020). In addition, the process of HDV RNA amplification is inseparable from the production of S-HDAg.

### Polymerase II Uses Genome Ribonucleoprotein as a Template to Transcribe mRNA

In nuclear, the parental gRNP migrates to Pol-II-containing speckles (Chang et al., 2008). Pol-II uses gRNP as a template to transcribe mRNA, which encodes the S-HDAg. HDV mRNA has a 5′ cap and a 3′ poly-A tail and migrates to the cytoplasm through a cell region maintenance 1-independent pathway (Macnaughton and Lai, 2002). Then mRNA is translated to S-HDAg in cytoplasm. Mediated by CCS and NLS, S-HDAg oligomerizes and re-enters the nucleus through the nuclear pore complex (Xia et al., 1992).

### Polymerase I Uses Genome Ribonucleoprotein as a Template to Replicate Antigenome RNA

After enough mRNA are transcribed, parental gRNP migrates to the nucleolus for agRNA replication. Although the migration machinery is not yet clear, it seems to be related to the newly synthesized S-HDAg (Macnaughton and Lai, 2006). Subsequently, Pol-I in the nucleolus uses the parental gRNP as a template, through the rolling-circle mechanism (Negro et al., 1989), to replicate linear agRNA polymers that are complementary to the gRNA. HDV antigenome strand ribozyme cleaves linear agRNA monomers from the linear agRNA polymers (Wadkins et al., 1999). Then, linear agRNA monomers are self-ligated into circular agRNA molecules. The self-ligation might be catalyzed by HDV RNA ribozyme or cell ligase (Reid and Lazinski, 2000).

### Polymerase II Uses Antigenome Ribonucleoprotein as a Template to Replicate Genome RNA

The structure of circular agRNA is similar to gRNA, it wraps around the newly synthesized HDAg multimers to form agRNP, and migrates to Pol-II-containing nuclear speckles. Pol-II uses agRNP as a template, through the rolling-circle mechanism, to replicate linear gRNA polymers. HDV genome strand ribozyme cleaves linear gRNA monomers from linear gRNA polymers. The linear gRNA monomers are self-ligated into circular gRNA molecules and wrap around the HDAg multimers to become the progeny gRNP.

According to the amplification process of HDV RNA, the progeny gRNP works as parental gRNP to amplify more mRNA or agRNA, or leaves the nucleus to form progeny virus.

## Small Hepatitis D Antigen Production

### Posttranslational Modification of Small Hepatitis D Antigen

As mentioned above, the amplification of HDV RNA is accompanied by the production of S-HDAg. Notably, after translation from mRNA, S-HDAg requires posttranslational modification, which is critical for its function. These critical modifications include: (i) Arginine methyltransferase catalyzes methylation at Arg-13, which is essential for RNA replication, affecting HDAg subcellular localization (Li et al., 2004); (ii) Acetyltransferase p300 catalyzes acetylation at Lys-72, which modulates HDAg subcellular localization and may be involved in viral RNA nucleocytoplasmic shuttling and replication (Mu et al., 2004); (iii) Extracellular signal-related kinases 1 and 2 catalyze phosphorylation at Ser-177, which mediates RNA replication and RNA edition (mention in 3.5.1) (Mu et al., 2001; Chen et al., 2008); (iv) Sumoylation at multiple lysine residues, enhancing RNA replication (Tseng et al., 2010).

### Function of Small Hepatitis D Antigen

After binding to HDV RNA, S-HDAg further interacts with several components of the host cell: (i) Pol-II. S-HDAg interacts with 9/12 subunits of Pol-II (Cao et al., 2009), the combination and loosening between S-HDAg and Pol-II clamp accelerate RNA elongation speed at the cost of fidelity (Yamaguchi et al., 2007). Except that, S-HDAg displaces negative elongation factor to promote elongation (Yamaguchi et al., 2001); (ii) Histone. S-HDAg N-terminal 2–67 aa bind to histone H1e central globular domain, and H1e plays a significant role in HDV replication (Lee and Sheu, 2008); (iii) Transcription factor. S-HDAg RBD and a GA/GK-rich region interact with transcription factor Yin Yang 1, and further bind to Yin Yang 1 associated acetyltransferases CBP and p300, these factor and transferases enhance HDV RNA replication (Huang et al., 2008); (iv) Nucleolar phosphoprotein. N-terminal domain of both forms of HDAg interacts with nucleolar phosphoprotein B23 (Huang et al., 2001) and nucleolin (Lee et al., 1998) to target nucleolus and modulate HDV replication; (v) Chromatin remodeling factor. S-HDAg contains a short linear interacting motif KacXXR, which binds with a chromatin remodeling factor, bromodomain adjacent to zinc finger domain 2B protein, to activate Pol-II-dependent HDV RNA amplification and sustain HDV replication (Abeywickrama-Samarakoon et al., 2020).

In the Pol-II-dependent mRNA and gRNA amplification, S-HDAg mainly activates RNA amplification and promotes RNA elongation. In the Pol-I dependent agRNA replication, S-HDAg stabilizes the agRNA, protects it from nuclease decomposition, and promotes its cleavage and self-ligation (Harichandran et al., 2019).

## Large Hepatitis D Antigen Production

Hepatitis delta virus RNA amplification requires S-HDAg, while L-HDAg prevents HDV RNA amplification and is essential for virus assembly. After accumulating some S-HDAg in cells, HDV RNA will be edited to produce L-HDAg. The newly synthesized L-HDAg is also posttranslational modified.

### RNA Editing

**FIGURE 4:**
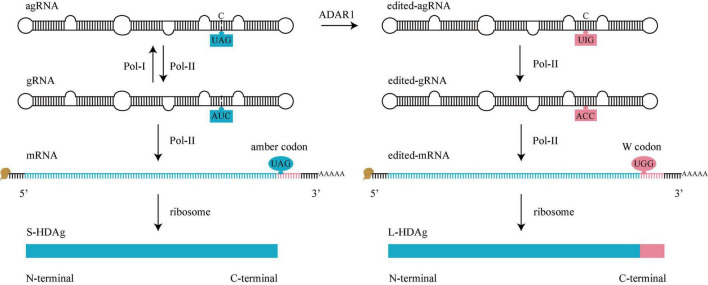
HDV RNA editing. Before editing (left), there is an amber codon (UAG, blue oval) at amber/W site of mRNA, translation will stop here to produce S-HDAg. During editing, adenosine deaminases acting on RNA 1 (ADAR1) converts UAG of agRNA to UIG (right). When Pol-II use these edited-agRNA as a template to replicate edited-gRNA, and use edited-gRNA as a template to transcribe edited-mRNA, there is a W codon (UGG, pink oval) at amber/W site of edited-mRNA, the translation won’t stop until next termination codon is met, producing an L-HDAg. gRNA, genomic RNA. agRNA, antigenomic RNA.

A family of proteins called adenosine deaminases acting on RNA (ADAR) catalyzes the adenosine (A) to inosine (I) editing reaction on dsRNA (Bass, 2002). HDV RNA editing is catalyzed by Adenosine deaminases acting on RNA 1 (ADAR1), which converts UAG triplet of agRNA into UIG triplet, and the original agRNA is converted into edited-agRNA (Figure 4). Before editing, the adenosine (A) mismatch with a cytosine (C), the editing converts the A-C mismatch pair into I-C match pair, which is a stabler structure. Currently, there is no known method to reverse this editing during infection (Casey, 2012). Presumably, the newly synthesized agRNA is available to be edited only before it binds to HDAg (Sato et al., 2004).

When Pol-II uses the edited-agRNA as a template, it replicates an ACC triplet on edited-gRNA. Pol-II further uses the edited-gRNA as a template to transcribe a W codon (UGG) on edited-mRNA. The edited-gRNA is defective for edited-agRNA replication (Sheu, 2002; Figure 4).

As mentioned above, there is a variable amber/W site in mRNA. For original mRNA, the site contains an amber codon (UAG, termination codon) and the translation stops here to produce an S-HDAg; For edited-mRNA, the site contains a W codon (UGG, encodes tryptophan), the translation doesn’t stop here, keeps adding the C-terminal 19 amino acids of L-HDAg until the next termination codon (Casey, 2012).

Adenosine deaminases acting on RNA 1 regulates the HDV life cycle by converting the early stage of HDV RNA amplification (mediated by S-HDAg) into the later stage of virus assembly (mediated by L-HDAg). Some genotypes of HDV (for example, HDV-6 and 8) do not display an A-C mismatch pair at the editing site, thereby use alternative transient conformation to increase the binding efficiency of ADAR1 (Dziri et al., 2021).

### Posttranslational Modification of Large Hepatitis D Antigen

Phosphorylation, acetylation, methylation, and sumoylation are posttranslational modifications of S-HDAg that also occur in the N-terminal identity region (1–195 aa) of L-HDAg. Additionally, the C-terminal extension region (196–214 aa) of L-HDAg requires farnesylation. The last four amino acids at the C-terminal (211–214 aa) of L-HDAg are Cys-Arg/Thr-Pro/Gln-Gln (C-R/T-P/Q-Q), which constitute a farnesylation signal (Huang et al., 2006). The farnesyl lipid group is added covalently to Cys-211 by the host’s farnesyl transferase, generating farnesylated L-HDAg.

Nuclear localization signal drives both farnesylated and non-farnesylated L-HDAg to enter into nucleus through the nuclear pore complex and the positively charged RBD drive them bind to HDV RNA. Nonetheless, the farnesyl group on L-HDAg is necessary for the combination between HDV RNP and HBsAg-containing envelope (Hwang and Lai, 1993), and is also required in HDV RNA amplification inhibition (Sato et al., 2004).

### Large Hepatitis D Antigen Drives Hepatitis Delta Virus Ribonucleoprotein Leave the Nucleus

After the start of RNA editing, the amount of L-HDAg in the nucleus increases in a time-dependent manner until the balance between the two HDAg is achieved. A small amount of L-HDAg is sufficient to inhibit the replication of gRNA. When L-HDAg exceeds S-HDAg, it inhibits the amplification of agRNA and mRNA (Modahl and Lai, 2000).

As the amount of L-HDAg increases in the nucleus, the proportion of L-HDAg monomers on the newly synthesized HDV gRNP is also increased. Considering the number of S-HDAg and L-HDAg are equivalent in circulating HDV (Bergmann and Gerin, 1986), we speculate that when the number of two HDAg is almost equal in a single HDV RNP, L-HDAg NES becomes the dominant signal, driving the egress of HDV gRNP from the nucleus through the nuclear pore complex (Lee et al., 2001). S-HDAg and L-HDAg cannot leave the nucleus in the absence of HDV RNA, presumably because binding to the RNA is required to expose L-HDAg NES (Tavanez et al., 2002).

Experiments have shown that despite the binding to HDAg, agRNA cannot leave the nucleus, most of the agRNA remains in the nucleus, but the amount of HDV gRNA in the cytoplasm and nucleus is almost equal (Macnaughton and Lai, 2002). However, edited-gRNA is exported from the nucleus and is eventually detected in the patient’s plasma (Homs et al., 2016; Sopena et al., 2018; Dziri et al., 2021). After leaving the nucleus, driven by farnesyl group on L-HDAg, HDV gRNP and edited-gRNP (but not agRNP) anchor to the endoplasmic reticulum membrane, where HBsAg is produced (Sureau and Negro, 2016).

## Assembly and Secretion

After assembling with HBsAg-containing envelope, HDV is secreted *via* HBV budding mechanism. As helper virus, HBV only mediates HDV *de novo* entry and secretion.

### Assembly and Secretion of Hepatitis B Virus in Hepatitis B Virus Mono-Infection

In HBV mono-infection, HBsAg is overexpressed in the endoplasmic reticulum and partially buds to the multivesicular body to bind to the HBV core particles (Niklasch et al., 2021). Mature HBV particles contain an equimolar proportion of the three forms of HBsAg (Sureau et al., 1993) and are secreted through the endosomal sorting complex required for transport-dependently multivesicular body (ESCRT/MVB) pathway (Lambert et al., 2007; Watanabe et al., 2007). The remaining excess of HBsAg is dimerized by protein disulfide isomerase in the endoplasmic reticulum compartment, from where it migrates to the pre-Golgi membrane as transmembrane dimer and self-assembles to form subviral particle (SVP) (Patient et al., 2007). These SVPs are 22 nm diameter spheres, composed of S-HBsAg and small amount of M-HBsAg, and do not contain viral genome (Sureau et al., 1993). After assembly, they are secreted through the Golgi apparatus classic constitutive secretory pathway (Patient et al., 2007). Since there is no L-HBsAg in sphere-SVPs, they cannot compete with HBV for hNTCP receptors. *Via* ESCRT/MVB pathway, Some HBsAg self-assembles to form filament-SVPs, which contain a much larger amount of the L-HBsAg compared to sphere-SVPs, and are secreted through ESCRT/MVB pathway like infectious viral particles (Jiang et al., 2015). The titer of SVP (10^12^–10^13^/mL) in serum of chronic hepatitis B patients is much higher than infectious HBV particle (10^8^–10^9^/mL) (Sureau and Negro, 2016), which is considered a strategy for HBV to evade the immune response.

### Assembly and Secretion of Hepatitis Delta Virus in Hepatitis B Virus and Hepatitis Delta Virus Dual-Infection

The envelope composition of HDV is similar to sphere-SVPs, S-HBsAg accounts for about 94%, and M-HBsAg accounts for about 5%. However, unlike sphere-SVPs that do not contain L-HBsAg, HDV envelope contains about 1% L-HBsAg (Bonino et al., 1986). A small number of L-HBsAg (about 3-4/virus) is sufficient for HDV infectivity (Le Duff et al., 2009). At the onset of acute infection, the titer of HDV is 10^10^–10^11^/mL serum (Sureau and Negro, 2016). Considering that the titer of circulating HDV is higher than HBV, approaching of SVPs, and the composition of HDV envelope is similar to sphere-SVPs, it could be speculated that HDV uses the sphere-SVPs secretion pathway, Golgi apparatus classic constitutive secretory pathway for its secretion.

When HDV RNP combines with HBsAg, the assembly signal of HDV RNP is located at the L-HDAg C-terminal farnesyl group (O’Malley and Lazinski, 2005), and the assembly signal of HBsAg is located at the TRM on the S region C-terminal matrix domain (Komla-Soukha and Sureau, 2006). The aromatic residues W196, W199, and W201, essential for TRM assembly capacity, are conserved in the envelope protein of the orthohepadnavirus (Komla-Soukha and Sureau, 2006). Owing to evolutionary conservation, other orthohepadnaviruses [such as woodchuck hepatitis virus (Lukash et al., 2019), the woolly monkey HBV (Barrera et al., 2004), and the tent-making bat HBV (Drexler et al., 2013)] also provide helper functions to HDV experimentally, except the envelope protein of duck hepatitis B virus as it lacks an HDV matrix domain (Sureau and Negro, 2016).

Without M-HBsAg and L-HBsAg expression, S-HBsAg drives HDV assembly and secretion, but the HDV only enveloped by S-HBsAg is not infectious (Sureau et al., 1993). L-HBsAg drives infection, and S-HBsAg drives assembly and secretion, while M-HBsAg may be dispensable (Sureau et al., 1994). Therefore, the infectivity of CHD patient’s serum may not be proportional solely to its HDV titer, but also affected by the number of L-HBsAg on HDV (Sureau et al., 1993). Except for the number of HDV and L-HBsAg, the genotypes of HBV and HDV also affect the infectivity. *In vitro* tests have shown that the genotype of HBV determines the number of HDV infected cells and the number of HDV genomes that successfully start replication after entry (Freitas et al., 2014). In addition, the combination of different HBV and HDV genotypes determine the efficacy of HDV egress and entry (Wang et al., 2021).

After co-transfecting plasmids encoding HDV and E1E2 (hepatitis C virus (HCV) envelope protein) into HCV host cells, HDV replicates and binds to E1E2, then is secreted and enters into other cells *via* E1E2 (Perez-Vargas et al., 2019). But clinically, HDV proliferation can’t rely on HCV instead of HBV, there are very few HBV DNA-negative, HCV RNA-positive, and HDV RNA-positive patients (Chemin et al., 2021; Roggenbach et al., 2021). These few cases could be ascribed to occult HBV infection (Raimondo et al., 2019).

### Persisting of Hepatitis Delta Virus in Hepatitis Delta Virus Mono-Infection

Without HBsAg expression, HDV persists in humanized mice for at least 6 weeks, leading to host cell detachment and death due to rapid virus replication, or be converted by HBV infection to develop coinfection (Giersch et al., 2014). Cell death may lead to cell lysis with exposure of cell debris and newly synthesized HDV RNP, but the unenveloped HDV RNP cannot invade the host cell. Except for disappearance and conversion, HDV spreads through hepatocyte proliferation (Giersch et al., 2019). Recently, we have discovered HDV-like agents in several taxa (termite, fish, toad, newt, snake, bird, woodchuck, rat, bat, and deer), and these agents lack the association with a respective animal hepadnavirus (Wille et al., 2018; Chang et al., 2019; Hetzel et al., 2019; Paraskevopoulou et al., 2020; Bergner et al., 2021; Iwamoto et al., 2021). This implies that HDV may not be a satellite virus initially, and the adaptation of HDV to HBsAg was an occasional and recent evolutionary event that provides HDV hepatotropism and accelerates the spread of HDV in humans (Netter et al., 2021; Zhang and Urban, 2021).

**FIGURE 5:**
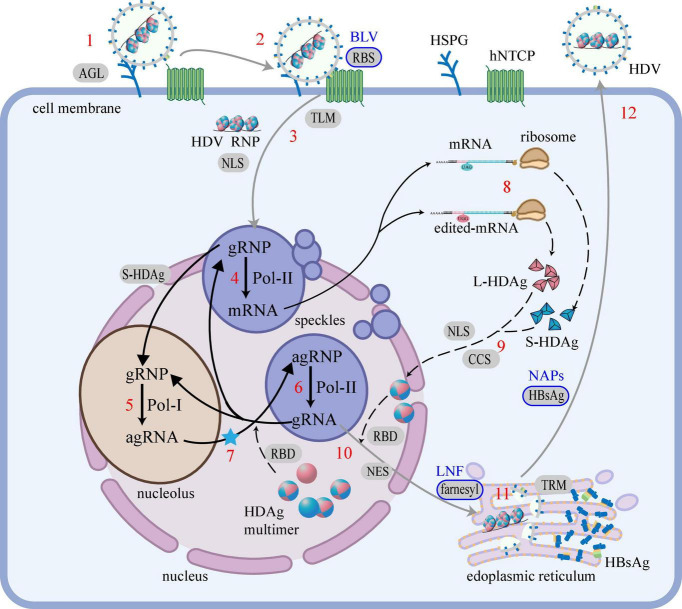
HDV life cycle and the regions that drive each step. The life cycle of HDV is mainly divided into 5 steps: (i) *De novo* entry, (1) attachment; (2) recognition; (3) entry into cells; (ii) RNA amplification, (4) mRNA transcription; (5) agRNA replication; (6) gRNA replication; (7) RNA editing (blue star); (iii) HDAg production, (8) translation; (9) modification; (iv) Assembly, (10) HDV RNP assembly; (11) HDV particles assembly; (v) Secretion, (12) secretion. Black words mark cell or virus components, red numbers mark life cycle steps, words on the gray background mark the regions that drive the step and the blue border marks the regions that have been applicated to HDV therapy, blue words mark the corresponding therapies. The gray line represents the migration of the virus, the solid black line represents the migration of the RNA, and the black dashed line represents the migration of the protein. Regions of HDAg: RBD, RNA-binding domain; CCS, Coiled-coil sequence; NLS, Nuclear localization signal; NES, Nuclear export signal; farnesyl, farnesyl lipid group. Regions of HBsAg: RBS, Receptor-binding site; TLM, Translocation motif; AGL, Antigenic loop; TRM, Try-rich motif. Cell or virus components: HSPG, heparan sulfate proteoglycans; hNTCP, human sodium taurocholate coreceptor peptide; gRNA, genomic RNA; agRNA, antigenomic RNA.

The entire life cycle of HDV (*de novo* entry, RNA amplification, HDAg production, assembly, and secretion) and the regions that drive each step are illustrated in Figure 5.

## Therapy of Chronic Hepatitis D

### Interferon-Immune Activator

Hepatitis delta virus replication triggers host cell to produce IFN-β and IFN-λ (Zhang et al., 2018), which are secreted out of the cell. Both secreted IFNs and administered IFNs are recognized by cognate receptors [such as interferon alpha receptor (IFNAR), interferon lambda receptor (IFNLR), and interleukin 10 receptor (IL10R)] on the surface of cells. The recognition induces IFN-stimulated genes, which inhibit viral replication, lower the cellular permissiveness, and activate the adaptive immune response (Zhang and Urban, 2020, 2021).

Interferon-α and Interferon-β belong to type I IFN, and they activate IFN-stimulated genes after being recognized by IFNAR1/IFNAR2, which are ubiquitously expressed on eukaryotic cells membrane (Sommereyns et al., 2008). Compared to standard IFN-α, pegylated IFN-α (Peg-IFN-α) has a longer plasma half-life, allowing a once-a-week administration, better compliance and efficiency. A meta-analysis showed that after administration of standard IFN-α for 12–48 weeks and 24–72 weeks of treatment-free follow-up, 17.4% of CHD patients had undetectable HDV RNA (Abbas et al., 2011); while after administration of Peg-IFN-α for 48 and 24 weeks of treatment-free follow-up, 31.0% of CHD patients had undetectable HDV RNA (Wedemeyer et al., 2011). Both standard IFN-α and Peg-IFN-α lead to frequent and sometimes severe adverse reactions, such as flu-like symptoms, asthenia, weight loss, alopecia, thrombocytopenia, and leukopenia (Abbas et al., 2011; Wedemeyer et al., 2011).

Interferon-λ is a type III IFN that activates IFN-stimulated genes after being recognized by IFNLR1/IL10R2, which are preferentially expressed in the epithelial tissues of the lung, liver, and gut (Sommereyns et al., 2008). The specificity of receptor expression imparts IFN-λ improved liver targeting and higher tolerability than IFN-α, resulting in fewer systemic adverse reactions and comparable antiviral efficacy. The common on-treatment adverse reactions included flu-like symptoms and elevated transaminase levels (Etzion et al., 2019). In the humanized mouse model, standard IFN-λ effectively reduced all intrahepatic markers of HDV infection (Giersch et al., 2017). After administration of Peg-IFN-λ for 48 and 24 weeks of treatment-free follow-up, 35.7% of CHD patients had undetectable HDV RNA (Etzion et al., 2019).

### Bulevirtide (BLV)-Competing With Large Hepatitis B Surface Antigen Receptor-Binding Site to Inhibit Entry

Bulevirtide (formerly known as Myrcludex B) is a myristoylated synthetic lipopeptide, with the same amino acids sequence as the N-terminal 47 aa in L-HBsAg RBS. *Via* binding to hNTCP, Bulevirtide competes with L-HBsAg RBS and inhibits the entry of HBV and HDV into hepatocytes. The effect of Bulevirtide monotherapy is not satisfactory, after administration of Bulevirtide for 48 weeks, only 13.3% of CHD patients had undetectable HDV RNA. However, the effect of administrating Bulevirtide with other drugs is excellent. When Bulevirtide and Peg-IFN-α are administered in combination for 48 weeks, 80.0% of CHD patients had undetectable HDV RNA, and after an additional 24 weeks of treatment-free follow-up, still, 53.3% of CHD patients had undetectable HDV RNA. Hitherto, Bulevirtide is administered daily by subcutaneous injections; oral preparations are still under development. The drug is generally well-tolerated, and the most frequent adverse reactions are increased total bile salts (asymptomatic and reversible after the treatment is discontinued) and injection site reactions (Kang and Syed, 2020).

### Lonafarnib (LNF)-Preventing Large Hepatitis D Antigen Farnesylation and Inhibiting Assembly

Lonafarnib is a farnesyl transferase inhibitor that prevents the farnesylation of L-HDAg, thus blocking the combination between HDV RNP and HBsAg. Lonafarnib should be administered with other antiviral drugs to achieve the best effect. When Lonafarnib and Ritonavir (RTV) are administered in combination for 24 weeks, 38.5% of CHD patients had undetectable HDV RNA (Lok et al., 2021). When Lonafarnib, Ritonavir, and Peg-IFN-λ are administered in combination for 24 weeks, 50.0% of CHD patients had undetectable HDV RNA, and after another 24 weeks of treatment-free follow-up, 22.7% of CHD patients still had undetectable HDV RNA. Lonafarnib is associated with significant adverse reactions, such as vomiting, diarrhea, infection, nausea, decreased appetite, fatigue, and upper respiratory tract infections (Dhillon, 2021).

### Nucleic Acid Polymers (NAPs)-Inhibiting Hepatitis B Surface Antigen Secretion

Nucleic acid polymers (NAPs) are phosphorothioated oligonucleotides, which means in every phosphodiester linkage of the oligonucleotides, one of non-bridging oxygen atom is replaced by a sulfur atom. In local hydrophobic microenvironment, the free electron in the non-bridging sulfur atom can transit from the unshared (charged) state to a shared (uncharged) state with the central phosphorous atom. The transition ability of the sulfur atom allows NAPs to adopt a localized hydrophobic property along their long axis and form a hydrophobic surface, the rest part of the NAPs forms a hydrophilic surface, altogether confer NAPs amphipathic property (Vaillant, 2019).

The host protein interaction with NAPs that drives their antiviral effects is not yet clear. Current study suggests *via* folding together of the hydrophobic surfaces, amphipathic NAPs bind amphipathic α-helices in class I viral glycoproteins, which are present on the surface of many enveloped viruses (Blanchet et al., 2019). This may explain why NAPs possess broad spectrum antiviral ability (Vaillant et al., 2006; Cardin et al., 2009). NAPs inhibit secretion of HBsAg from hepatocytes and block replenishment of HBsAg in the circulation, allowing host mediated clearance. Unlike nucleoside/nucleotide analogs that possess antiviral activity in HBV infection but are not effective against HDV infection (Yurdaydin et al., 2008; Abbas et al., 2016), NAPs are effective therapy for HDV infection.

REP 2139-Ca as a kind of NAPs has been administrated to CHD patients in the REP301 and REP 301-LTF study. After administration of REP 2139-Ca for 15 weeks, followed by a combination of REP 2139-Ca and Peg-IFN-α for 15 weeks, 83.3% of CHD patients had undetectable HDV RNA. Continuing to administrate Peg-IFN-α for 33 weeks, 75.0% of CHD patients had undetectable HDV RNA (Bazinet et al., 2017). After another 18 months of treatment-free follow-up, 58.3% of CHD patients still had undetectable HDV RNA (Bazinet et al., 2018). The most frequent adverse reactions observed during REP 2139-Ca monotherapy were pyrexia and chills, and less frequently conjunctival hyperemia, headache, and asthenia near the end of infusion (Bazinet et al., 2017).

The detail clinical data of the above-mentioned four types of drugs are summarized in Table 2.

**TABLE 2:** Comparison of the effects of different drugs on HDV.

Drug(s)	Does of the first drug	Administrated period (week)	Follow-up period (week)	HDV RNA negative rate (%)	Sample number	Adverse reactions
IFN-α	Meta*^a^*	12–48	24–72	17.4	92	Frequent and serious
Peg-IFN-α	180 μg qw	48	24	31.0	29	
Peg-IFN-λ	180 μg qw	48	24	35.7	14	Fewer systemic
BLV	2 mg qd	48	0	13.3	15	Asymptomatic and reversible
BLV + Peg-IFN-α	2 mg qd	48	0	80.0	15	
BLV + Peg-IFN-α	2 mg qd	48	24	53.3	15	
LNF + RTV	50 mg bid	24	0	38.5	13	Significant
LNF + RTV + Peg-IFN-λ	50 mg bid	24	0	50.0	22	
LNF + RTV + Peg-IFN-λ	50 mg bid	24	24	22.7	22	
NAPs + Peg-IFN-α*^b^*	500/250 mg qd*^b^*	30*^b^*	0	83.3	12	Not serious
NAPs + Peg-IFN-α*^b^*	500/250 mg qd*^b^*	30*^b^*	33*^c^*	75.0	12	
NAPs + Peg-IFN-α*^b^*	500/250 mg qd*^b^*	30*^b^*	111*^d^*	58.3	12	

*^a^Meta-analysis, different doses are administered in different experiments; ^b^Administered REP 2139-Ca (a kind of NAPs) 500 mg qd for 15 weeks, followed by a combination of REP 2139-Ca 250 mg qd and Peg-IFN-α 180 ug qd for 15 weeks; ^c^Continued to administrate Peg-IFN-α; ^d^The first 33 weeks of which continued to administrate Peg-IFN-α. IFN, Interferon; Peg, Pegylated; BLV, Bulevirtide; LNF, Lonafarnib; RTV, Ritonavir; NAPs, Nucleic acid polymers; qw, once weekly; qd, once daily; bid, twice daily.*

## Conclusion and Prospects

Hepatitis delta virus is a robust pathogen that spreads widely. Since an ideal therapy is yet unavailable, in-depth exploration is essential. The present review summarizes the effects of the specific regions of HDAg and HBsAg on HDV life cycle: (i) *De novo* entry. After attaching to HSPG *via* AGL, HDV is recognized by hNTCP *via* RBS to enter into hepatocyte. In cytoplasm, HDV RNP is released *via* TLM, and enters into nucleus *via* NLS; (ii) RNA amplification. S-HDAg activates the amplification of mRNA and gRNA, stabilizes agRNA and promotes its cleavage and self-ligation; (iii) HDAg production. After modification, mediated by CCS and NLS, HDAg oligomerizes and re-enters the nucleus; (iv) Assembly. HDV RNA wraps around HDAg multimers to form HDV gRNP *via* RBD, NES drives the egress of HDV gRNP from the nucleus. After leaving the nucleus, HDV gRNP anchors to the endoplasmic reticulum membrane *via* farnesyl group, and binds to HBsAg-containing envelope *via* the interaction between TRM and farnesyl group to form HDV; (v) Secretion. Driven by HBsAg-containing envelope, HDV secretes *via* the Golgi apparatus classic constitutive secretory pathway.

Some regions have been applicated for HDV therapy: (i) RBS: Bulevirtide is an L-HBsAg RBS analog, competing with RBS for specific receptor hNTCP to block HDV entry; (ii) Farnesyl group: Lonafarnib is a farnesyl transferase inhibitor, preventing the farnesylation of L-HDAg, thus blocking the combination between HDV RNP and HBsAg; (iii) HBsAg: NAPs are phosphorothioated oligonucleotides that bind to amphipathic α-helices of HBsAg, preventing the secretion of HBsAg. Except these three regions, several other regions are potential therapeutic targets. For example, blocking NLS or NES of HDAg will prevent HDAg nucleocytoplasmic shuttling, blocking CCS or RBD of HDAg will prevent the assembly among HDAg or between HDAg and RNA, and blocking TRM of HBsAg will prevent the assembly between HDV RNP and HBsAg-containing envelope.

There are still several mechanisms underlying the HDV life cycle are yet ill-understood, including but not limited to the following: the assembly structure of HDV RNA and HDAg; similarities and differences between HBV and HDV during the *de novo* entry; key factors driving HDV to remove the HBsAg-containing envelope and expose HDV RNP; method to change the HDV RNP dominant signal from farnesyl group (anchor to endoplasmic reticulum) in assembly stage to NLS (enter into the nucleus) in new round of the *de novo* entry; whether and how Pol-III participates in the amplification of HDV RNA; the domains or signals of S-HDAg involved in the migration of gRNP from the nucleoplasm to the nucleolus during RNA amplification; enzymes catalyzing the connection of HDV RNA linear monomers to form circular monomers. Investigating HDV proliferation mechanisms and the functions of these specific regions on HDAg and HBsAg will help us to discover new treatment strategies and develop new drugs associated with better therapeutic effects, fewer adverse reactions, and lower recurrence rates.

## Author Contributions

JZ: data curation, formal analysis, writing-original draft, and writing-review and editing. XG: data curation, writing-original draft, and writing-review and editing. JD: writing-review and editing. HX: formal analysis, validation, and writing-review and editing. JN: funding acquisition, supervision, and writing-review and editing. XC: data curation, supervision, validation, and writing-review and editing. All authors contributed to the article and approved the submitted version.

## Conflict of Interest

The authors declare that the research was conducted in the absence of any commercial or financial relationships that could be construed as a potential conflict of interest.

## Publisher’s Note

All claims expressed in this article are solely those of the authors and do not necessarily represent those of their affiliated organizations, or those of the publisher, the editors and the reviewers. Any product that may be evaluated in this article, or claim that may be made by its manufacturer, is not guaranteed or endorsed by the publisher.

## References

[B1] Abbas Z. Khan M. A. Salih M. Jafri W. (2011). Interferon alpha for chronic hepatitis D. *Cochrane Database Syst. Rev.* 2011:Cd006002. 10.1002/14651858.CD006002.pub2 22161394 PMC6823236

[B2] Abbas Z. Memon M. S. Umer M. A. Abbas M. Shazi L. (2016). Co-treatment with pegylated interferon alfa-2a and entecavir for hepatitis D: a randomized trial. *World J. Hepatol.* 8 625–631. 10.4254/wjh.v8.i14.625 27190579 PMC4867420

[B3] Abeywickrama-Samarakoon N. Cortay J. C. Sureau C. Müller S. Alfaiate D. Guerrieri F. (2020). Hepatitis Delta virus histone mimicry drives the recruitment of chromatin remodelers for viral RNA replication. *Nat. Commun.* 11:419. 10.1038/s41467-020-14299-9 31964889 PMC6972770

[B4] Barrera A. Guerra B. Lee H. Lanford R. E. (2004). Analysis of host range phenotypes of primate hepadnaviruses by in vitro infections of hepatitis D virus pseudotypes. *J. Virol.* 78 5233–5243. 10.1128/jvi.78.10.5233-5243.2004 15113905 PMC400381

[B5] Bass B. L. (2002). RNA editing by adenosine deaminases that act on RNA. *Annu. Rev. Biochem.* 71 817–846. 10.1146/annurev.biochem.71.110601.135501 12045112 PMC1823043

[B6] Bazinet M. Pântea V. Cebotarescu V. Cojuhari L. Jimbei P. Albrecht J. (2017). Safety and efficacy of REP 2139 and pegylated interferon alfa-2a for treatment-naive patients with chronic hepatitis B virus and hepatitis D virus co-infection (REP 301 and REP 301-LTF): a non-randomised, open-label, phase 2 trial. *Lancet Gastroenterol. Hepatol.* 2 877–889. 10.1016/s2468-1253(17)30288-1 28964701

[B7] Bazinet M. Pantea V. Cebotarescu V. Cojuhari L. Jimbei P. Vaillant A. (2018). Establishment of persistent functional remission of HBV and HDV infection following REP 2139 and pegylated interferon alpha 2a therapy in patients with chronic HBV/HDV co-infection: 18 month follow-up results from the REP 301-LTF study. *J. Hepatol.* 68:S509. 10.1016/s0168-8278(18)31266-2

[B8] Bergmann K. F. Gerin J. L. (1986). Antigens of hepatitis delta virus in the liver and serum of humans and animals. *J. Infect. Dis.* 154 702–706. 10.1093/infdis/154.4.702 3745977

[B9] Bergner L. M. Orton R. J. Broos A. Tello C. Becker D. J. Carrera J. E. (2021). Diversification of mammalian deltaviruses by host shifting. *Proc. Natl. Acad. Sci. U S A.* 118:e2019907118. 10.1073/pnas.2019907118 33397804 PMC7826387

[B10] Blanchet M. Sinnathamby V. Vaillant A. Labonté P. (2019). Inhibition of HBsAg secretion by nucleic acid polymers in HepG2.2.15?cells. *Antiviral Res.* 164 97–105. 10.1016/j.antiviral.2019.02.009 30771404

[B11] Blanchet M. Sureau C. (2007). Infectivity determinants of the hepatitis B virus pre-S domain are confined to the N-terminal 75 amino acid residues. *J. Virol.* 81 5841–5849. 10.1128/jvi.00096-07 17376925 PMC1900317

[B12] Bonino F. Heermann K. H. Rizzetto M. Gerlich W. H. (1986). Hepatitis delta virus: protein composition of delta antigen and its hepatitis B virus-derived envelope. *J. Virol.* 58 945–950. 10.1128/jvi.58.3.945-950.1986 3701932 PMC253003

[B13] Bonino F. Hoyer B. Ford E. Shih J. W. Purcell R. H. Gerin J. L. (1981). The delta agent: HBsAg particles with delta antigen and RNA in the serum of an HBV carrier. *Hepatology* 1 127–131. 10.1002/hep.1840010207 6169614

[B14] Botelho-Souza L. F. Vasconcelos M. P. A. Dos Santos A. O. Salcedo J. M. V. Vieira D. S. (2017). Hepatitis delta: virological and clinical aspects. *Virol. J.* 14:177. 10.1186/s12985-017-0845-y 28903779 PMC5597996

[B15] Bruss V. Hagelstein J. Gerhardt E. Galle P. R. (1996). Myristylation of the large surface protein is required for hepatitis B virus in vitro infectivity. *Virology* 218 396–399. 10.1006/viro.1996.0209 8610467

[B16] Bulla G. A. Siddiqui A. (1989). Negative regulation of the hepatitis B virus pre-S1 promoter by internal DNA sequences. *Virology* 170 251–260. 10.1016/0042-6822(89)90373-5 2718384

[B17] Cao D. Haussecker D. Huang Y. Kay M. A. (2009). Combined proteomic-RNAi screen for host factors involved in human hepatitis delta virus replication. *RNA* 15 1971–1979. 10.1261/rna.1782209 19776158 PMC2764473

[B18] Cardin R. D. Bravo F. J. Sewell A. P. Cummins J. Flamand L. Juteau J. M. (2009). Amphipathic DNA polymers exhibit antiviral activity against systemic murine cytomegalovirus infection. *Virol. J.* 6:214. 10.1186/1743-422x-6-214 19954538 PMC2794273

[B19] Casey J. L. (2012). Control of ADAR1 editing of hepatitis delta virus RNAs. *Curr. Top. Microbiol. Immunol.* 353 123–143. 10.1007/82_2011_146 21732238 PMC3572862

[B20] Chang J. Nie X. Chang H. E. Han Z. Taylor J. (2008). Transcription of hepatitis delta virus RNA by RNA polymerase II. *J. Virol.* 82 1118–1127. 10.1128/jvi.01758-07 18032511 PMC2224410

[B21] Chang W. S. Pettersson J. H. Le Lay C. Shi M. Lo N. Wille M. (2019). Novel hepatitis D-like agents in vertebrates and invertebrates. *Virus Evol.* 5:vez021. 10.1093/ve/vez021 31321078 PMC6628682

[B22] Chemin I. Pujol F. H. Scholtès C. Loureiro C. L. Amirache F. Levrero M. (2021). Preliminary evidence for hepatitis delta virus exposure in patients who are apparently not infected with hepatitis B virus. *Hepatology* 73 861–864. 10.1002/hep.31453 32628280 PMC7898870

[B23] Chen Y. S. Huang W. H. Hong S. Y. Tsay Y. G. Chen P. J. (2008). ERK1/2-mediated phosphorylation of small hepatitis delta antigen at serine 177 enhances hepatitis delta virus antigenomic RNA replication. *J. Virol.* 82 9345–9358. 10.1128/jvi.00656-08 18632853 PMC2546944

[B24] Dhillon S. (2021). Lonafarnib: first approval. *Drugs* 81 283–289. 10.1007/s40265-020-01464-z 33590450 PMC7985116

[B25] Drexler J. F. Geipel A. König A. Corman V. M. van Riel D. Leijten L. M. (2013). Bats carry pathogenic hepadnaviruses antigenically related to hepatitis B virus and capable of infecting human hepatocytes. *Proc. Natl. Acad. Sci. U S A.* 110 16151–16156. 10.1073/pnas.1308049110 24043818 PMC3791787

[B26] Dziri S. Rodriguez C. Gerber A. Brichler S. Alloui C. Roulot D. (2021). Variable in vivo hepatitis D Virus (HDV) RNA editing rates according to the HDV genotype. *Viruses* 13:1572. 10.3390/v13081572 34452437 PMC8402866

[B27] Etzion O. Hamid S. S. Lurie Y. Gane E. Bader N. Yardeni D. (2019). End of study results from LIMT HDV study: 36% durable virologic response at 24 weeks post-treatment with pegylated interferon lambda monotherapy in patients with chronic hepatitis delta virus infection. *J. Hepatol.* 70 E32–E32. 10.1016/s0618-8278(19)30058-1

[B28] Fattovich G. Giustina G. Christensen E. Pantalena M. Zagni I. Realdi G. (2000). Influence of hepatitis delta virus infection on morbidity and mortality in compensated cirrhosis type B the European concerted action on viral hepatitis (Eurohep). *Gut* 46 420–426. 10.1136/gut.46.3.420 10673308 PMC1727859

[B29] Freitas N. Abe K. Cunha C. Menne S. Gudima S. O. (2014). Support of the infectivity of hepatitis delta virus particles by the envelope proteins of different genotypes of hepatitis B virus. *J. Virol.* 88 6255–6267. 10.1128/jvi.00346-14 24648462 PMC4093875

[B30] Fukano K. Tsukuda S. Oshima M. Suzuki R. Aizaki H. Ohki M. (2018). Troglitazone impedes the oligomerization of sodium taurocholate cotransporting polypeptide and entry of hepatitis B virus into hepatocytes. *Front. Microbiol.* 9:3257. 10.3389/fmicb.2018.03257 30671048 PMC6331526

[B31] Giersch K. Bhadra O. D. Volz T. Allweiss L. Riecken K. Fehse B. (2019). Hepatitis delta virus persists during liver regeneration and is amplified through cell division both in vitro and in vivo. *Gut* 68 150–157. 10.1136/gutjnl-2017-314713 29217749

[B32] Giersch K. Helbig M. Volz T. Allweiss L. Mancke L. V. Lohse A. W. (2014). Persistent hepatitis D virus mono-infection in humanized mice is efficiently converted by hepatitis B virus to a productive co-infection. *J. Hepatol.* 60 538–544. 10.1016/j.jhep.2013.11.010 24280293

[B33] Giersch K. Homs M. Volz T. Helbig M. Allweiss L. Lohse A. W. (2017). Both interferon alpha and lambda can reduce all intrahepatic HDV infection markers in HBV/HDV infected humanized mice. *Sci. Rep.* 7:3757. 10.1038/s41598-017-03946-9 28623307 PMC5473824

[B34] Glenn J. S. White J. M. (1991). trans-dominant inhibition of human hepatitis delta virus genome replication. *J. Virol.* 65 2357–2361. 10.1128/jvi.65.5.2357-2361.1991 2016764 PMC240587

[B35] Greco-Stewart V. S. Schissel E. Pelchat M. (2009). The hepatitis delta virus RNA genome interacts with the human RNA polymerases I and III. *Virology* 386 12–15. 10.1016/j.virol.2009.02.007 19246067

[B36] Griffin B. Chasovskikh S. Dritschilo A. Casey J. (2014). Hepatitis delta antigen requires a flexible quasi-double-stranded RNA structure to bind and condense hepatitis delta virus RNA in a ribonucleoprotein complex. *J. Virol.* 88 7402–7411. 10.1128/jvi.00443-14 24741096 PMC4054418

[B37] Gudima S. Chang J. Moraleda G. Azvolinsky A. Taylor J. (2002). Parameters of human hepatitis delta virus genome replication: the quantity, quality, and intracellular distribution of viral proteins and RNA. *J. Virol.* 76 3709–3719. 10.1128/jvi.76.8.3709-3719.2002 11907210 PMC136113

[B38] Gudima S. Meier A. Dunbrack R. Taylor J. Bruss V. (2007). Two potentially important elements of the hepatitis B virus large envelope protein are dispensable for the infectivity of hepatitis delta virus. *J. Virol.* 81 4343–4347. 10.1128/jvi.02478-06 17251287 PMC1866104

[B39] Harichandran K. Shen Y. Stephenson Tsoris S. Lee S. C. Casey J. L. (2019). Hepatitis delta antigen regulates mRNA and antigenome RNA levels during hepatitis delta virus replication. *J. Virol.* 93 e1989–e1918. 10.1128/jvi.01989-18 30728256 PMC6450126

[B40] Herrscher C. Pastor F. Burlaud-Gaillard J. Dumans A. Seigneuret F. Moreau A. (2020). Hepatitis B virus entry into HepG2-NTCP cells requires clathrin-mediated endocytosis. *Cell Microbiol.* 22:e13205. 10.1111/cmi.13205 32216005

[B41] Hetzel U. Szirovicza L. Smura T. Prähauser B. Vapalahti O. Kipar A. (2019). Identification of a novel deltavirus in boa constrictors. *mBio* 10:e00014–e19. 10.1128/mBio.00014-19 30940697 PMC6445931

[B42] Homs M. Rodriguez-Frias F. Gregori J. Ruiz A. Reimundo P. Casillas R. (2016). Evidence of an exponential decay pattern of the hepatitis delta virus evolution rate and fluctuations in quasispecies complexity in long-term studies of chronic delta infection. *PLoS One* 11:e0158557. 10.1371/journal.pone.0158557 27362848 PMC4928832

[B43] Hsu C. W. Juang H. H. Kuo C. Y. Li H. P. Iang S. B. Lin S. H. (2019). Structural pattern differences in unbranched rod-like RNA of hepatitis delta virus affect RNA editing. *Viruses* 11;934. 10.3390/v11100934 31614652 PMC6832723

[B44] Hu Q. Zhang F. Duan L. Wang B. Ye Y. Li P. (2020). E-cadherin plays a role in hepatitis B virus entry through affecting glycosylated sodium-taurocholate cotransporting polypeptide distribution. *Front. Cell. Infect. Microbiol.* 10:74. 10.3389/fcimb.2020.00074 32175289 PMC7056903

[B45] Huang W. H. Chen C. W. Wu H. L. Chen P. J. (2006). Post-translational modification of delta antigen of hepatitis D virus. *Curr. Top. Microbiol. Immunol.* 307 91–112. 10.1007/3-540-29802-9_5 16903222

[B46] Huang W. H. Mai R. T. Lee Y. H. (2008). Transcription factor YY1 and its associated acetyltransferases CBP and p300 interact with hepatitis delta antigens and modulate hepatitis delta virus RNA replication. *J. Virol.* 82 7313–7324. 10.1128/jvi.02581-07 18480431 PMC2493304

[B47] Huang W. H. Yung B. Y. Syu W. J. Lee Y. H. (2001). The nucleolar phosphoprotein B23 interacts with hepatitis delta antigens and modulates the hepatitis delta virus RNA replication. *J. Biol. Chem.* 276 25166–25175. 10.1074/jbc.M010087200 11309377

[B48] Hwang S. B. Lai M. M. (1993). Isoprenylation mediates direct protein-protein interactions between hepatitis large delta antigen and hepatitis B virus surface antigen. *J. Virol.* 67 7659–7662. 10.1128/jvi.67.12.7659-7662.1993 8230486 PMC238236

[B49] Iwamoto M. Saso W. Nishioka K. Ohashi H. Sugiyama R. Ryo A. (2020). The machinery for endocytosis of epidermal growth factor receptor coordinates the transport of incoming hepatitis B virus to the endosomal network. *J. Biol. Chem.* 295 800–807. 10.1074/jbc.AC119.010366 31836663 PMC6970923

[B50] Iwamoto M. Saso W. Sugiyama R. Ishii K. Ohki M. Nagamori S. (2019). Epidermal growth factor receptor is a host-entry cofactor triggering hepatitis B virus internalization. *Proc. Natl. Acad. Sci. U S A.* 116 8487–8492. 10.1073/pnas.1811064116 30952782 PMC6486715

[B51] Iwamoto M. Shibata Y. Kawasaki J. Kojima S. Li Y. T. Iwami S. (2021). Identification of novel avian and mammalian deltaviruses provides new insights into deltavirus evolution. *Virus Evol.* 7:veab003. 10.1093/ve/veab003 33614159 PMC7882216

[B52] Jiang B. Hildt E. (2020). Intracellular trafficking of HBV particles. *Cells* 9:2023. 10.3390/cells9092023 32887393 PMC7563130

[B53] Jiang B. Himmelsbach K. Ren H. Boller K. Hildt E. (2015). Subviral hepatitis B virus filaments, like infectious viral particles, are released via multivesicular bodies. *J. Virol.* 90 3330–3341. 10.1128/jvi.03109-15 26719264 PMC4794700

[B54] Kang C. Syed Y. Y. (2020). Bulevirtide: first approval. *Drugs* 80 1601–1605. 10.1007/s40265-020-01400-1 32926353

[B55] Komla-Soukha I. Sureau C. (2006). A tryptophan-rich motif in the carboxyl terminus of the small envelope protein of hepatitis B virus is central to the assembly of hepatitis delta virus particles. *J. Virol.* 80 4648–4655. 10.1128/jvi.80.10.4648-4655.2006 16641257 PMC1472050

[B56] Lambert C. Döring T. Prange R. (2007). Hepatitis B virus maturation is sensitive to functional inhibition of ESCRT-III, Vps4, and gamma 2-adaptin. *J. Virol.* 81 9050–9060. 10.1128/jvi.00479-07 17553870 PMC1951427

[B57] Le Duff Y. Blanchet M. Sureau C. (2009). The pre-S1 and antigenic loop infectivity determinants of the hepatitis B virus envelope proteins are functionally independent. *J. Virol.* 83 12443–12451. 10.1128/jvi.01594-09 19759159 PMC2786703

[B58] Lee C. H. Chang S. C. Chen C. J. Chang M. F. (1998). The nucleolin binding activity of hepatitis delta antigen is associated with nucleolus targeting. *J. Biol. Chem.* 273 7650–7656. 10.1074/jbc.273.13.7650 9516470

[B59] Lee C. H. Chang S. C. Wu C. H. Chang M. F. (2001). A novel chromosome region maintenance 1-independent nuclear export signal of the large form of hepatitis delta antigen that is required for the viral assembly. *J. Biol. Chem.* 276 8142–8148. 10.1074/jbc.M004477200 11076934

[B60] Lee C. Z. Lin J. H. Chao M. McKnight K. Lai M. M. (1993). RNA-binding activity of hepatitis delta antigen involves two arginine-rich motifs and is required for hepatitis delta virus RNA replication. *J. Virol.* 67 2221–2227. 10.1128/jvi.67.4.2221-2227.1993 8445729 PMC240345

[B61] Lee C. Z. Sheu J. C. (2008). Histone H1e interacts with small hepatitis delta antigen and affects hepatitis delta virus replication. *Virology* 375 197–204. 10.1016/j.virol.2008.02.003 18314153

[B62] Lempp F. Ni Y. Urban S. (2016). Hepatitis delta virus: insights into a peculiar pathogen and novel treatment options. *Nat. Rev. Gastroenterol. Hepatol.* 13 580–589. 10.1038/nrgastro.2016.126 27534692

[B63] Lempp F. A. Wiedtke E. Qu B. Roques P. Chemin I. Vondran F. W. R. (2017). Sodium taurocholate cotransporting polypeptide is the limiting host factor of hepatitis B virus infection in macaque and pig hepatocytes. *Hepatology* 66 703–716. 10.1002/hep.29112 28195359

[B64] Li Y. J. Stallcup M. R. Lai M. M. (2004). Hepatitis delta virus antigen is methylated at arginine residues, and methylation regulates subcellular localization and RNA replication. *J. Virol.* 78 13325–13334. 10.1128/jvi.78.23.13325-13334.2004 15542683 PMC524986

[B65] Löffler-Mary H. Dumortier J. Klentsch-Zimmer C. Prange R. (2000). Hepatitis B virus assembly is sensitive to changes in the cytosolic S loop of the envelope proteins. *Virology* 270 358–367. 10.1006/viro.2000.0268 10792995

[B66] Lok A. S. Negro F. Asselah T. Farci P. Rizzetto M. (2021). Endpoints and new options for treatment of chronic hepatitis D. *Hepatology* 74 3479–3485. 10.1002/hep.32082 34331781 PMC9293075

[B67] Lukash T. Freitas N. Menne S. Gudima S. O. (2019). Down-regulation of hepatitis delta virus super-infection in the woodchuck model. *Virology* 531 100–113. 10.1016/j.virol.2019.03.002 30856482 PMC6486404

[B68] Macnaughton T. B. Lai M. M. (2002). Genomic but not antigenomic hepatitis delta virus RNA is preferentially exported from the nucleus immediately after synthesis and processing. *J. Virol.* 76 3928–3935. 10.1128/jvi.76.8.3928-3935.2002 11907232 PMC136093

[B69] Macnaughton T. B. Lai M. M. (2006). HDV RNA replication: ancient relic or primer? *Curr. Top. Microbiol. Immunol.* 307 25–45. 10.1007/3-540-29802-9_2 16903219

[B70] Macovei A. Petrareanu C. Lazar C. Florian P. Branza-Nichita N. (2013). Regulation of hepatitis B virus infection by Rab5, Rab7, and the endolysosomal compartment. *J. Virol.* 87 6415–6427. 10.1128/jvi.00393-13 23536683 PMC3648082

[B71] Meier A. Mehrle S. Weiss T. S. Mier W. Urban S. (2013). Myristoylated PreS1-domain of the hepatitis B virus L-protein mediates specific binding to differentiated hepatocytes. *Hepatology* 58 31–42. 10.1002/hep.26181 23213046

[B72] Modahl L. E. Lai M. M. (2000). The large delta antigen of hepatitis delta virus potently inhibits genomic but not antigenomic RNA synthesis: a mechanism enabling initiation of viral replication. *J. Virol.* 74 7375–7380. 10.1128/jvi.74.16.7375-7380.2000 10906190 PMC112257

[B73] Mu J. J. Chen D. S. Chen P. J. (2001). The conserved serine 177 in the delta antigen of hepatitis delta virus is one putative phosphorylation site and is required for efficient viral RNA replication. *J. Virol.* 75 9087–9095. 10.1128/jvi.75.19.9087-9095.2001 11533172 PMC114477

[B74] Mu J. J. Tsay Y. G. Juan L. J. Fu T. F. Huang W. H. Chen D. S. (2004). The small delta antigen of hepatitis delta virus is an acetylated protein and acetylation of lysine 72 may influence its cellular localization and viral RNA synthesis. *Virology* 319 60–70. 10.1016/j.virol.2003.10.024 14967488

[B75] Negro F. Korba B. E. Forzani B. Baroudy B. M. Brown T. L. Gerin J. L. (1989). Hepatitis delta virus (HDV) and woodchuck hepatitis virus (WHV) nucleic acids in tissues of HDV-infected chronic WHV carrier woodchucks. *J. Virol.* 63 1612–1618. 10.1128/jvi.63.4.1612-1618.1989 2926865 PMC248403

[B76] Netter H. J. Barrios M. H. Littlejohn M. Yuen L. K. W. (2021). Hepatitis delta virus (HDV) and delta-like agents: insights into their origin. *Front. Microbiol.* 12:652962. 10.3389/fmicb.2021.652962 34234753 PMC8256844

[B77] Ni Y. Lempp F. A. Mehrle S. Nkongolo S. Kaufman C. Fälth M. (2014). Hepatitis B and D viruses exploit sodium taurocholate co-transporting polypeptide for species-specific entry into hepatocytes. *Gastroenterology* 146 1070–1083. 10.1053/j.gastro.2013.12.024 24361467

[B78] Niklasch M. Zimmermann P. Nassal M. (2021). The hepatitis B virus nucleocapsid-dynamic compartment for infectious virus production and new antiviral target. *Biomedicines* 9:1577. 10.3390/biomedicines9111577 34829806 PMC8615760

[B79] O’Malley B. Lazinski D. W. (2005). Roles of carboxyl-terminal and farnesylated residues in the functions of the large hepatitis delta antigen. *J. Virol.* 79 1142–1153. 10.1128/jvi.79.2.1142-1153.2005 15613342 PMC538544

[B80] Paraskevopoulou S. Pirzer F. Goldmann N. Schmid J. Corman V. M. Gottula L. T. (2020). Mammalian deltavirus without hepadnavirus coinfection in the neotropical rodent Proechimys semispinosus. *Proc. Natl. Acad. Sci. U S A.* 117 17977–17983. 10.1073/pnas.2006750117 32651267 PMC7395443

[B81] Patient R. Hourioux C. Sizaret P. Y. Trassard S. Sureau C. Roingeard P. (2007). Hepatitis B virus subviral envelope particle morphogenesis and intracellular trafficking. *J. Virol.* 81 3842–3851. 10.1128/jvi.02741-06 17267490 PMC1866106

[B82] Perez-Vargas J. Amirache F. Boson B. Mialon C. Freitas N. Sureau C. (2019). Enveloped viruses distinct from HBV induce dissemination of hepatitis D virus in vivo. *Nat. Commun.* 10:2098. 10.1038/s41467-019-10117-z 31068585 PMC6506506

[B83] Poisson F. Roingeard P. Baillou A. Dubois F. Bonelli F. Calogero R. A. (1993). Characterization of RNA-binding domains of hepatitis delta antigen. *J. Gen. Virol.* 74(Pt 11) 2473–2478. 10.1099/0022-1317-74-11-2473 8245865

[B84] Polaris Observatory Collaborators (2018). Global prevalence, treatment, and prevention of hepatitis B virus infection in 2016: a modelling study. *Lancet. Gastroenterol. Hepatol.* 3 383–403. 10.1016/s2468-1253(18)30056-6 29599078

[B85] Polo J. M. Jeng K. S. Lim B. Govindarajan S. Hofman F. Sangiorgi F. (1995). Transgenic mice support replication of hepatitis delta virus RNA in multiple tissues, particularly in skeletal muscle. *J. Virol.* 69 4880–4887. 10.1128/jvi.69.8.4880-4887.1995 7609056 PMC189302

[B86] Rabe B. Glebe D. Kann M. (2006). Lipid-mediated introduction of hepatitis B virus capsids into nonsusceptible cells allows highly efficient replication and facilitates the study of early infection events. *J. Virol.* 80 5465–5473. 10.1128/jvi.02303-05 16699026 PMC1472160

[B87] Raimondo G. Locarnini S. Pollicino T. Levrero M. Zoulim F. Lok A. S. (2019). Update of the statements on biology and clinical impact of occult hepatitis B virus infection. *J. Hepatol.* 71 397–408. 10.1016/j.jhep.2019.03.034 31004683

[B88] Reid C. E. Lazinski D. W. (2000). A host-specific function is required for ligation of a wide variety of ribozyme-processed RNAs. *Proc. Natl. Acad. Sci. U S A.* 97 424–429. 10.1073/pnas.97.1.424 10618434 PMC26679

[B89] Riccitelli N. Lupták A. (2013). HDV family of self-cleaving ribozymes. *Prog. Mol. Biol. Transl. Sci.* 120 123–171. 10.1016/b978-0-12-381286-5.00004-4 24156943

[B90] Roggenbach I. Chi X. Lempp F. A. Qu B. Walter L. Wu R. (2021). HDV seroprevalence in HBsAg-positive patients in china occurs in hotspots and is not associated with HCV mono-infection. *Viruses* 13:1799. 10.3390/v13091799 34578380 PMC8473203

[B91] Ryu W. Netter H. Bayer M. Taylor J. (1993). Ribonucleoprotein complexes of hepatitis delta virus. *J. Virol.* 67 3281–3287. 10.1128/jvi.67.6.3281-3287.1993 8497052 PMC237669

[B92] Sato S. Cornillez-Ty C. Lazinski D. W. (2004). By inhibiting replication, the large hepatitis delta antigen can indirectly regulate amber/W editing and its own expression. *J. Virol.* 78 8120–8134. 10.1128/jvi.78.15.8120-8134.2004 15254184 PMC446097

[B93] Sheu G. T. (2002). Initiation of hepatitis delta virus (HDV) replication: HDV RNA encoding the large delta antigen cannot replicate. *J. Gen. Virol.* 83(Pt 10) 2507–2513. 10.1099/0022-1317-83-10-2507 12237434

[B94] Siddiqui A. Jameel S. Mapoles J. (1986). Transcriptional control elements of hepatitis B surface antigen gene. *Proc. Natl. Acad. Sci. U S A.* 83 566–570. 10.1073/pnas.83.3.566 3456153 PMC322904

[B95] Sommereyns C. Paul S. Staeheli P. Michiels T. (2008). IFN-lambda (IFN-lambda) is expressed in a tissue-dependent fashion and primarily acts on epithelial cells in vivo. *PLoS Pathog.* 4:e1000017. 10.1371/journal.ppat.1000017 18369468 PMC2265414

[B96] Sopena S. Godoy C. Tabernero D. Homs M. Gregori J. Riveiro-Barciela M. (2018). Quantitative characterization of hepatitis delta virus genome edition by next-generation sequencing. *Virus Res.* 243 52–59. 10.1016/j.virusres.2017.10.003 28988126

[B97] Steiner S. Huebner M. T. Dreesman G. R. (1974). Major polar lipids of hepatitis B antigen preparations: evidence for the presence of a glycosphingolipid. *J. Virol.* 14 572–577. 10.1128/jvi.14.3.572-577.1974 4853069 PMC355551

[B98] Stockdale A. Kreuels B. Henrion M. Giorgi E. Kyomuhangi I. de Martel C. (2020). The global prevalence of hepatitis D virus infection: systematic review and meta-analysis. *J. Hepatol.* 73 523–532. 10.1016/j.jhep.2020.04.008 32335166 PMC7438974

[B99] Stoeckl L. Funk A. Kopitzki A. Brandenburg B. Oess S. Will H. (2006). Identification of a structural motif crucial for infectivity of hepatitis B viruses. *Proc. Natl. Acad. Sci. U S A.* 103 6730–6734. 10.1073/pnas.0509765103 16618937 PMC1458949

[B100] Sureau C. Guerra B. Lanford R. E. (1993). Role of the large hepatitis B virus envelope protein in infectivity of the hepatitis delta virion. *J. Virol.* 67 366–372. 10.1128/jvi.67.1.366-372.1993 8416375 PMC237372

[B101] Sureau C. Guerra B. Lee H. (1994). The middle hepatitis B virus envelope protein is not necessary for infectivity of hepatitis delta virus. *J. Virol.* 68 4063–4066. 10.1128/jvi.68.6.4063-4066.1994 8189544 PMC236918

[B102] Sureau C. Negro F. (2016). The hepatitis delta virus: Replication and pathogenesis. *J. Hepatol.* 64 S102–S116. 10.1016/j.jhep.2016.02.013 27084031

[B103] Sureau C. Salisse J. (2013). A conformational heparan sulfate binding site essential to infectivity overlaps with the conserved hepatitis B virus a-determinant. *Hepatology* 57 985–994. 10.1002/hep.26125 23161433

[B104] Tavanez J. P. Cunha C. Silva M. C. David E. Monjardino J. Carmo-Fonseca M. (2002). Hepatitis delta virus ribonucleoproteins shuttle between the nucleus and the cytoplasm. *RNA* 8 637–646. 10.1017/s1355838202026432 12022230 PMC1370284

[B105] Taylor J. (2015). Hepatitis D virus replication. *Cold Spring Harb. Perspect. Med.* 5:a021568. 10.1101/cshperspect.a021568 26525452 PMC4632862

[B106] Tseng C. H. Cheng T. S. Shu C. Y. Jeng K. S. Lai M. M. (2010). Modification of small hepatitis delta virus antigen by SUMO protein. *J. Virol.* 84 918–927. 10.1128/jvi.01034-09 19889771 PMC2798338

[B107] Vaillant A. (2019). REP 2139: antiviral mechanisms and applications in achieving functional control of HBV and HDV infection. *ACS Infect. Dis.* 5 675–687. 10.1021/acsinfecdis.8b00156 30199230

[B108] Vaillant A. Juteau J. M. Lu H. Liu S. Lackman-Smith C. Ptak R. (2006). Phosphorothioate oligonucleotides inhibit human immunodeficiency virus type 1 fusion by blocking gp41 core formation. *Antimicrob. Agents Chemother.* 50 1393–1401. 10.1128/aac.50.4.1393-1401.2006 16569857 PMC1426958

[B109] Verrier E. R. Colpitts C. C. Bach C. Heydmann L. Weiss A. Renaud M. (2016). A targeted functional RNA interference screen uncovers glypican 5 as an entry factor for hepatitis B and D viruses. *Hepatology* 63 35–48. 10.1002/hep.28013 26224662

[B110] Wadkins T. S. Perrotta A. T. Ferré-D’Amaré A. R. Doudna J. A. Been M. D. (1999). A nested double pseudoknot is required for self-cleavage activity of both the genomic and antigenomic hepatitis delta virus ribozymes. *RNA* 5 720–727. 10.1017/s1355838299990209 10376872 PMC1369799

[B111] Wang W. Lempp F. A. Schlund F. Walter L. Decker C. C. Zhang Z. (2021). Assembly and infection efficacy of hepatitis B virus surface protein exchanges in 8 hepatitis D virus genotype isolates. *J. Hepatol.* 75 311–323. 10.1016/j.jhep.2021.03.025 33845061

[B112] Watanabe T. Sorensen E. M. Naito A. Schott M. Kim S. Ahlquist P. (2007). Involvement of host cellular multivesicular body functions in hepatitis B virus budding. *Proc. Natl. Acad. Sci. U S A.* 104 10205–10210. 10.1073/pnas.0704000104 17551004 PMC1891263

[B113] Wedemeyer H. Yurdaydìn C. Dalekos G. N. Erhardt A. Çakaloğlu Y. Değertekin H. (2011). Peginterferon plus adefovir versus either drug alone for hepatitis delta. *N. Engl. J. Med.* 364 322–331. 10.1056/NEJMoa0912696 21268724

[B114] Wille M. Netter H. J. Littlejohn M. Yuen L. Shi M. Eden J. S. (2018). A divergent hepatitis D-like agent in birds. *Viruses* 10:720. 10.3390/v10120720 30562970 PMC6315422

[B115] Xia Y. Chang M. Wei D. Govindarajan S. Lai M. (1990). Heterogeneity of hepatitis delta antigen. *Virology* 178 331–336. 10.1016/0042-6822(90)90415-n 2389557

[B116] Xia Y. P. Lai M. M. (1992). Oligomerization of hepatitis delta antigen is required for both the trans-activating and trans-dominant inhibitory activities of the delta antigen. *J. Virol.* 66 6641–6648. 10.1128/jvi.66.11.6641-6648.1992 1404608 PMC240160

[B117] Xia Y. P. Yeh C. T. Ou J. H. Lai M. M. (1992). Characterization of nuclear targeting signal of hepatitis delta antigen: nuclear transport as a protein complex. *J. Virol.* 66 914–921. 10.1128/jvi.66.2.914-921.1992 1731113 PMC240792

[B118] Yamaguchi Y. Filipovska J. Yano K. Furuya A. Inukai N. Narita T. (2001). Stimulation of RNA polymerase II elongation by hepatitis delta antigen. *Science* 293 124–127. 10.1126/science.1057925 11387440

[B119] Yamaguchi Y. Mura T. Chanarat S. Okamoto S. Handa H. (2007). Hepatitis delta antigen binds to the clamp of RNA polymerase II and affects transcriptional fidelity. *Genes Cells* 12 863–875. 10.1111/j.1365-2443.2007.01094.x 17584298

[B120] Yan H. Zhong G. Xu G. He W. Jing Z. Gao Z. (2012). Sodium taurocholate cotransporting polypeptide is a functional receptor for human hepatitis B and D virus. *Elife* 1:e00049. 10.7554/eLife.00049 PMC3485615 23150796

[B121] Yang S. Shen Z. Kang Y. Sun L. Viswanathan U. Guo H. (2021). A putative amphipathic alpha helix in hepatitis B virus small envelope protein plays a critical role in the morphogenesis of subviral particles. *J. Virol.* 95 e2399–e2320. 10.1128/jvi.02399-20 33536177 PMC8103704

[B122] Yurdaydin C. Bozkaya H. Onder F. O. Sentürk H. Karaaslan H. Akdoğan M. (2008). Treatment of chronic delta hepatitis with lamivudine vs lamivudine + interferon vs interferon. *J. Viral. Hepat.* 15 314–321. 10.1111/j.1365-2893.2007.00936.x 18307594

[B123] Zhang Z. Filzmayer C. Ni Y. Sültmann H. Mutz P. Hiet M. S. (2018). Hepatitis D virus replication is sensed by MDA5 and induces IFN-β/λ responses in hepatocytes. *J. Hepatol.* 69 25–35. 10.1016/j.jhep.2018.02.021 29524530

[B124] Zhang Z. Urban S. (2020). Interplay between Hepatitis D Virus and the Interferon Response. *Vijruses* 12:1334. 10.3390/v12111334 33233762 PMC7699955

[B125] Zhang Z. Urban S. (2021). New insights into HDV persistence: The role of interferon response and implications for upcoming novel therapies. *J. Hepatol.* 74 686–699. 10.1016/j.jhep.2020.11.032 33276031

[B126] Zuccola H. J. Rozzelle J. E. Lemon S. M. Erickson B. W. Hogle J. M. (1998). Structural basis of the oligomerization of hepatitis delta antigen. *Structure* 6 821–830. 10.1016/s0969-2126(98)00084-7 9687364

